# Challenges and Burdens in the Coronary Artery Disease Care Pathway for Patients Undergoing Percutaneous Coronary Intervention: A Contemporary Narrative Review

**DOI:** 10.3390/ijerph20095633

**Published:** 2023-04-25

**Authors:** Monika Kodeboina, Kerstin Piayda, Inge Jenniskens, Pearl Vyas, Sara Chen, Ramon Julian Pesigan, Nicole Ferko, Barkha P. Patel, Annamaria Dobrin, Jayson Habib, Jennifer Franke

**Affiliations:** 1 Cardiovascular Center Aalst, OLV Clinic, 9300 Aalst, Belgium; monika211@gmail.com; 2 Department of Advanced Biomedical Sciences, University of Naples Federico II, 80138 Naples, Italy; 3 Clinic for Internal Medicine and Cardiology, Marien Hospital, 52066 Aachen, Germany; 4 Cardiovascular Center Frankfurt, 60389 Frankfurt, Germany; k.piayda@cvcfrankfurt.de; 5 Department of Cardiology and Vascular Medicine, Medical Faculty, Justus-Liebig-University Giessen, 35392 Giessen, Germany; 6 Philips, 5684 PC Best, The Netherlands; inge.jenniskens@philips.com (I.J.); pearl.vyas@philips.com (P.V.); ramonjulian.pesigan@philips.com (R.J.P.); 7 Philips, San Diego, CA 92130, USA; sara.chen@philips.com; 8 EVERSANA, Burlington, ON L7N 3H8, Canada; nicole.ferko@eversana.com (N.F.); barkha.patel@eversana.com (B.P.P.); annamaria.dobrin@eversana.com (A.D.); habib.jayson@gmail.com (J.H.); 9 Philips Chief Medical Office, 22335 Hamburg, Germany

**Keywords:** coronary artery disease, treatment, percutaneous coronary intervention, echocardiography, myocardial ischemia, diagnostic imaging, integration, automation, inefficiencies, burdens

## Abstract

Clinical and economic burdens exist within the coronary artery disease (CAD) care pathway despite advances in diagnosis and treatment and the increasing utilization of percutaneous coronary intervention (PCI). However, research presenting a comprehensive assessment of the challenges across this pathway is scarce. This contemporary review identifies relevant studies related to inefficiencies in the diagnosis, treatment, and management of CAD, including clinician, patient, and economic burdens. Studies demonstrating the benefits of integration and automation within the catheterization laboratory and across the CAD care pathway were also included. Most studies were published in the last 5–10 years and focused on North America and Europe. The review demonstrated multiple potentially avoidable inefficiencies, with a focus on access, appropriate use, conduct, and follow-up related to PCI. Inefficiencies included misdiagnosis, delays in emergency care, suboptimal testing, longer procedure times, risk of recurrent cardiac events, incomplete treatment, and challenges accessing and adhering to post-acute care. Across the CAD pathway, this review revealed that high clinician burnout, complex technologies, radiation, and contrast media exposure, amongst others, negatively impact workflow and patient care. Potential solutions include greater integration and interoperability between technologies and systems, improved standardization, and increased automation to reduce burdens in CAD and improve patient outcomes.

## 1. Introduction

Coronary artery disease (CAD) is the leading cause of mortality, affecting approximately 1.72% of the global population, resulting in 9 million deaths per year [1,2]. The total CAD prevalence for adults was estimated to be 7.1% in the U.S. from 2017 to 2020 [3] and 5.11% in 2019 in Europe [4], respectively, and its incidence is expected to rise [1]. Atherosclerosis, an inflammatory disease affecting the arteries, is the primary pathological process leading to CAD and can be caused by multiple risk factors, including age, obesity, smoking, and poor diet [1,5]. Two distinct clinicopathological entities exist for CAD: chronic coronary syndrome (CCS) and acute coronary syndrome (ACS) [6]. CCS can result from microvascular disease and/or restricted blood flow generated through the chronic, progressive growth of plaque into a vessel’s lumen. Although the development of ACS is a complex process, it is usually a consequence of plaque disruption in coronary arteries characterized by a sudden blockage of blood flow to the heart muscles [7,8]. Depending on the level of severity, ACS can be further classified as ST-elevation myocardial infarction (STEMI), non-ST elevation myocardial infarction (NSTEMI), or unstable angina [9,10]. 

The CAD care pathway involves multiple steps, including primary prevention, emergency care, diagnosis, treatment, secondary prevention, and follow-up (Table 1). Early risk assessment helps to establish preventative measures, including lifestyle modifications and cholesterol-lowering therapies. Secondary prevention aims to prevent further damage and disease progression, including myocardial infarction (MI) events, which may lead to percutaneous coronary intervention (PCI) and coronary bypass graft surgery (CABG) as treatment strategies [11].

Percutaneous coronary intervention (PCI) is a minimally invasive, nonsurgical technique that aims to relieve narrowing or occlusion of the coronary artery through the insertion and inflation of a small balloon catheter or a stent (over the balloon). The material is advanced via vascular arterial access in the groin or wrist and expands and deploys in position to relieve the stenosis [12]. Clinical indications that require PCI include acute STEMI, NSTEMI, unstable and stable angina (with at least intermediate pretest probability for coronary artery disease and positive noninvasive stress-testing or high pretest probability), and any critical coronary artery stenosis not qualifying for coronary artery bypass grafting [12] with symptoms and/or prognostic significance. PCI is performed in the heart catheterization (“cath”) lab, which is prone to operational inefficiencies [13,14], distractions [15], and closed-loop communication [16]. Late arrival times, procedural delays, and inadequate staff result in negative effects for patient care [17]. 

Numerous innovative technologies have emerged for the management of CAD to help support clinical decision-making. Examples include intravascular ultrasound (IVUS) and optical coherence tomography (OCT), two high-resolution intracoronary imaging modalities, and fractional flow reserve (FFR) and instantaneous wave-free ratio (iFR) to guide coronary revascularization. IVUS can precisely guide stent implantation at the index PCI (Class IIA) [18,19], while FFR and iFR measure the hemodynamic relevance of coronary stenosis (Class IA) [19,20]. In addition, technological advances that automate monitoring and encourage medication adherence are promising but are highly dependent on patient engagement [21]. 

Despite advancements, the high rate of CAD events underscores the complexity of CAD care. Access to innovative technologies and services remains problematic, and this problem has intensified during the COVID-19 pandemic [22]. To preserve limited resources for COVID-19 patients, many cardiac procedures were deferred [23]. A multicenter Spanish study observed a 40% reduction in STEMI patients presenting to the hospital during the pandemic [24]. Patients were afraid to seek acute care because they were frightened of becoming infected with COVID-19 during their hospital stay. Furthermore, a retrospective European study observed a 19% reduction in primary PCI procedures and delayed treatment, which may have contributed to the increase in mortality from 4.9 to 6.8% from 2019 to 2020 in patients with STEMI [25]. Inequitable access to technologies and variability in their use can negatively impact patients and healthcare workers. Relatedly, clinician burnout in cardiology is documented as a current burden that is intensifying [26,27]. 

In medicine, steps along the CAD care pathway have traditionally been viewed in isolation without close coordination that allows for continuity of care for the patient and overall efficiency for healthcare providers. As the management of patients with CAD involves interactions across multiple specialties and healthcare settings, this increases the risk of fragmented care [28]. Comprehensively addressing the burdens across the entire continuum of care acknowledges the connectedness of the care pathway and the need to optimize resources to deliver better solutions for patients and healthcare providers [29].

Furthermore, recent compilations of research have emphasized technological advancements; however, a summary of the challenges and inefficiencies across the CAD care pathway and the source of those burdens has been limited, particularly in relation to PCI. As PCI is increasingly utilized as a treatment of choice outside of medical therapy for CAD, the objective of this review is to explore the contemporary evidence on the challenges and unmet needs related to the diagnosis, treatment, and management of CAD. We especially focus on PCI and present evidence on the benefits of improved integration within the cardiac cath lab and across the CAD care pathway.

## 2. Methods

Literature review searches were conducted using PubMed and supplemented with grey Google searches to answer several research questions as needed (see Table 2, including used search terms). Two researchers reviewed titles and abstracts of all records identified, followed by a review of potentially relevant full texts to identify those that met predetermined inclusion criteria. As the review was “targeted” and not systematic, inclusion/exclusion criteria were applied differently across topic areas. A nonsystematic review was preferred to keep the scope achievable and focused, given the multiple research questions. To limit extensive searches, researchers focused on identifying systematic literature reviews (SLRs) or meta-analyses where appropriate. Most studies were published in the last 5–10 years, written in English, and focused on North America and Europe. Articles were generally excluded based on the following criteria: smaller study (sample size less than 50), niche focus (e.g., comparing different techniques, etc.), not CAD-focused, pediatrics, clinical trials/studies not serving the broader objective, and preclinical data. In case of uncertainty, both researchers reviewed the article and came to a conclusion on whether it should be included in this study or not. Over a dozen iterative searches with different search strings and term combinations were conducted, which produced a list of approximately 9000 titles to be screened and de-duplicated. Approximately 250 abstracts were retained for further screening, and grey literature searches were conducted for additional evidence points. 

## 3. Results

### 3.1. Primary Prevention

The key to early CAD detection is an appropriate risk assessment to determine if there is a relevant chance of coronary disease. Thorough risk assessments can help to establish preventative measures. Risk factors for CAD include but are not limited to age, sex, cholesterol levels, diabetes, obesity, high blood pressure, activity levels, and smoking status [30]. The first step in the CAD pathway is typically to complete a medical history, with a physical examination and blood tests. Primary prevention requires attention to risk factors beginning early in life, including a family history of premature atherosclerotic cardiovascular disease and chronic kidney disease [31]. Preventative measures include lifestyle modifications with a healthy diet, exercise, smoking cessation, and cholesterol-lowering. Statin therapy can help prevent cardiac events in those at intermediate and high risk [31]. In low-risk and asymptomatic patients, risk stratification is often determined with a family history, physical examination, and use of established modalities, including a validated risk score, an electrocardiogram (ECG), stress test, or coronary artery calcium score [32]. However, a study found that approximately half of the acute myocardial infarction patients in the high-risk cohort were unaware of and/or not considered to have a high cardiovascular risk, suggesting that prediction techniques and patient education can be further optimized [33]. As such, coronary artery calcium scoring via cardiac computed tomography angiography (CCTA) could help determine if statins and aspirin benefit intermediate-risk patients [32]. For symptomatic patients, establishing the probability of coronary occlusion will guide the choice of further diagnostic examinations, including ECG, stress testing, CCTA, and angiography [11].

### 3.2. CAD Diagnosis—Challenges with Access, Accuracy, and Appropriate Use

For patients with suspected CAD, the aim is to confirm the causality of symptoms. Patients are stratified based on risk, and then the need for, or timing of, revascularization is determined [6]. In terms of diagnostic modalities, functional and anatomical evaluations can be conducted to achieve these aims.

In the emergency department, acute chest pain is commonly misdiagnosed in specific patient populations, resulting in fatal outcomes. In more than 50% of fatal acute myocardial infarctions, patients die outside the hospital without receiving acute in-hospital treatment [34]. Common initial misdiagnoses include nonspecific chest pain, gastrointestinal disease, musculoskeletal pain, and arrhythmias. Reasons for missed acute myocardial infarctions have been reported to include incorrect ECG interpretation and failure to order appropriate diagnostic tests [35]. An extensive malpractice claims database analysis showed that most patients in cardiovascular outpatient general medicine malpractice cases have at least one risk factor suggestive of ischemic heart disease, such as hypertension, tobacco use, or prior cardiovascular disease [36]. Furthermore, the evidence shows that chest pain and ACS are more commonly misdiagnosed in women, with 5% of ACS misdiagnosed in women versus 3% in men (*p* < 0.001) [37]. As probable ACS was noted in 39% of women and 44.5% of men (*p* < 0.001), myocardial infarction is likely underestimated in women [37]. 

In patients with acute CAD, the timely delivery of care is critical for favorable outcomes [38]. Current practice guidelines recommend that patients receive an ECG within 10 min of presenting to an emergency department [39,40]. The ECG is the most used initial diagnostic test for patients with suspected ACS as it is inexpensive, widely available, and noninvasive [41]. Additionally, ECG changes of ischemia occur prior to infarction and can be detected, providing the ability for clinicians to intervene before myocardial cell death [41]. Noninvasive stress tests for inducible ischemia are recommended before an invasive strategy (Class IA) for suspected ACS with normal ECG results and stable troponin levels [42]. In patients with NSTEMI and unstable angina, a meta-analysis of randomized controlled trials found that early versus delayed invasive management (i.e., coronary angiography) was associated with a lower incidence of major adverse cardiovascular events (MACE) (relative risk (RR) 0.65, 95% confidence intervals (CI) 0.49–0.87; *p* = 0.003) and recurrent ischemia (RR 0.42, 95%CI 0.26–0.69; *p* < 0.0005) [43]. However, accessing emergent diagnostic care in ACS is often delayed due to several factors, such as suboptimal patient flow and access to appropriate technologies. A real-world analysis in U.S. adults with ACS revealed that emergency department adherence rates to national standards for arrival time-to-ECG read-out time and physician-ordered biomarker turnaround-time were only 42 and 37%, respectively, with delays related to patient complaints inconsistent with ACS, the timing of stress testing, and medication administration [38]. In Europe, specialized chest pain units (CPUs), compared to emergency departments, may improve access to care for patients with ACS. However, findings from the German CPU Registry showed that the median time from symptom onset to first medical contact was prolonged at 2 h in patients with STEMI and 4 h in patients with unstable angina and NSTEMI [44]. In addition, while ECGs were performed in nearly all patients, only 71% of patients obtained an ECG within the recommended 10 min [44]. These findings suggest that strategies, such as increasing access to reliable and timely diagnostic testing, show potential for improving ACS care and patient flow through the emergency department. Relatedly, weekend admission is associated with prolonged delays in accessing diagnostic care and increased mortality risk for NSTEMI and STEMI [45,46]. 

Activation and use of emergency medical services (EMS), including prehospital ECGs, can also impact the timeliness of care. An SLR and meta-analysis showed that prehospital ECGs were associated with a 7.0 min increase in scene arrival-to-hospital arrival time (i.e., a delay in EMS) and a 2.9 min increase in scene time for patients with STEMI versus without prehospital ECGs [47]. However, a mobile cloud-based 12-lead electrocardiogram (MC-ECG) transmission system has been shown to be useful for patients in rural areas with delays in time to treatment [48]. A study in Japan found that the length of hospital stay was significantly reduced for patients with STEMI when an MC-ECG transmission system was used for transport by EMS as opposed to the conventional method in which a physician checks the ECG in the hospital (12.0 days vs. 16.0 days; *p* = 0.039) [48]. If a patient with STEMI requires an interfacility transfer to a PCI center, a retrospective analysis found that the median ECG-to-EMS activation interval was 20 min, representing 32% of the overall emergency department length of stay. Notably, faster EMS activation was more likely to achieve a shorter emergency department length of stay [49]. 

For clinicians, selecting an appropriate diagnostic modality for patients with stable angina is based on patient factors, local availability and expertise, test performance, and cost [6]. Multiple publications support the initial use of exercise ECGs as a cost-effective option, followed by other diagnostic tests, such as stress echocardiography or cardiac magnetic resonance (CMR) [46]. However, there is significant uncertainty on how to come to a reliable diagnosis [6]. Functional testing (e.g., stress echocardiography, CMR), which has long been the test of choice to risk stratify patients with stable CAD, shows modest agreement with the CAD severity detected by anatomical investigations with invasive coronary angiography (ICA) but has shown to be ineffective in settings with a low prevalence of obstructive CAD [50,51,52]. Anatomical testing (e.g., CCTA) has unique benefits to guide primary prevention in younger patients presenting with suspected CAD. In patients with low-risk stable chest pain, the PROMISE trial demonstrated that anatomical strategies were less expensive and more effective compared to functional testing [53]. Research on the uses of anatomical and functional tests in various subpopulations is consistently expanding and beyond the scope of this review. The overall strengths and limitations of both functional and anatomical investigations are presented in Table 3.

The variability and complexity of diagnostic test choice by patient type can lead to unnecessary invasive testing in both patients with and without CAD, which is costly and potentially harmful [32]. Data from the U.S. CathPCI Registry showed that 39.2% of individuals without known CAD undergoing elective coronary angiography had no angiographic evidence of CAD (between 2004 and 2008) [57]. Relatedly, an observational study found that a quarter of all patients (308,083 of 1,225,562) undergoing elective diagnostic coronary angiography were asymptomatic [58]. However, greater adoption of the guidelines might improve diagnostic decision-making in patients with suspected CCS. Based on the EURECA Imaging Registry, adoption of the 2019 European Society of Cardiology (ESC) guidelines resulted in less frequent ICA, as well as greater diagnosis of obstructive CAD (60% vs. 39%; *p* < 0.001) and revascularization (54% vs. 37%; *p* < 0.001) [59]. Furthermore, the 2019-ESC-pretest probability model was found to be accurate in predicting obstructive stenosis detected by a combined endpoint of CCTA and ICA [60].

While ICA remains the gold standard for diagnosing obstructive CAD, it is associated with high cost, radiation exposure, patient discomfort, and procedure-related risks [61]. The assessment of CAD severity by ICA may be flawed because the angiographic severity of a given epicardial stenosis does not necessarily correspond with its functional significance [62]. Furthermore, researchers found that the diagnostic performance of single-photon emission computed tomography (SPECT), stress echocardiography, and ICA was generally poor when directly compared with FFR [63]. An incorrect indication for coronary angiography may, in turn, lead to inappropriate use of PCI, as observed from findings using the National Cardiovascular Data Registry [58]. 

Although the exercise ECG has been the foundation of CAD diagnostic testing for decades, the accuracy of treadmill stress ECGs has been documented at 60%, and false positives (i.e., exercise ECG changes with nonobstructive disease on anatomical testing) are common [64]. Data from the National Ambulatory Medical Care Survey and National Hospital Ambulatory Medical Care Survey from 2008 to 2010 showed that an estimated 13,710 of 571,755 patients with a falsely negative cardiac stress test without prior history of CAD will experience a cardiac event annually [65]. However, if patients were correctly diagnosed and treated, up to 2202 of these events were estimated to be preventable [65]. These false negative tests amount to USD 210.2 million in testing costs, USD 19.4 million in hospital costs for nonfatal myocardial infarction and stroke, and USD 89.0 million in lost productivity (a total of USD 318.6 million, excluding the downstream costs associated with premature death or other preventable cardiovascular events) [65]. 

While echocardiography is the most used cardiac imaging modality, its manual interpretation can be onerous and subject to human error [66,67]. A U.S. review found that manual analysis of echocardiograms is error-prone and suffers from high intra- and inter-reader variability [68,69]. Furthermore, trained cardiological experts well-equipped to analyze these images are often unavailable in low-resource settings, contributing to a significant provider burden [70]. 

Although improvements in diagnostic outcomes depend on the use of the right test in the right patient at the right time, evidence for how certain technologies can alleviate some diagnostic burdens is expanding. For example, for intermediate to high-risk patients with stable chest pain and no known CAD, CCTA is effective for diagnosing CAD, risk stratification, and guiding treatment decisions [40]. An SLR in patients with suspected CAD showed that, versus functional stress testing, CCTA is associated with an increased incidence of ICA, coronary revascularization, CAD diagnoses, and use of medical therapy, with a reduced incidence of myocardial infarction [71]. Furthermore, CCTA in patients with stable and unstable chest pain can reduce downstream resource use and costs without compromising patient satisfaction, morbidity, or mortality [72,73] and results in fewer and more appropriate PCIs, shortened emergency department stays, and lower MACE rates over five years [73,74,75]. Of note, the FFR_CT_ technique has been developed to reproduce the acquisition of functional information on top of a detailed anatomical atherosclerotic burden description with CT and is associated with substantial reductions in ICA use [76]. However, arrhythmia, increased heart rate, obesity, and calcification during CCTA may degrade the image quality, and the requirement for contrast dye can be limiting in certain patients [6]. Recent meta-analyses and real-world studies are exploring the impact that different testing strategies may have in helping to alleviate unnecessary or inappropriate downstream diagnostic testing in CAD and associated events, with results varying based on the comparators and subpopulations of focus [77,78].

### 3.3. CAD Revascularization—Challenges with Access, Appropriate Use, and Rising Patient Complexity

Coronary revascularization using PCI is an important therapeutic option when managing patients with CAD, and utilization has increased over time [79,80]. However, a study found that PCI, in addition to optimal medical therapy in patients with severe ischemic left ventricular systolic dysfunction, did not result in a lower incidence of death or hospitalization for heart failure versus optimal medical therapy alone [81]. This underscores the importance of using PCI in the appropriate patient population. Of note, data from the U.S. NIS from 2010 to 2014 reported that the 30-day hospital cost of PCI is approximately USD 10.8 billion per year [82], representing a significant financial burden to the U.S. healthcare system. As a result, it is also critical to determine which patients will optimally benefit from PCI to avoid an unnecessary economic burden.

Patients presenting with STEMI should be treated with primary PCI with drug-eluting stent implantation within 120 min from the first medical contact [7,83] or 90 min from patient presentation to the first balloon inflation. However, many patients with STEMI transferred to PCI centers from the emergency department do not receive PCI within these timeframes [49,84], often due to poor coordination between EMS agencies and PCI centers [49]. Furthermore, the direct admission of patients with STEMI to the cath lab significantly decreased mortality by shortening pain-to-balloon time (160 min vs. 240 min) and door-to-balloon (D2B) time (35 min vs. 75 min) versus emergency department admissions [85]. Longer D2B times in PCI are associated with poorer patient outcomes, including higher rates of mortality and MACE [86,87,88], which are associated with substantial costs. The average total cost incurred per patient during the first MACE (defined as hospitalization for myocardial infarction or stroke) was USD 19,642 for patients initially diagnosed with NSTEMI (38.8%), unstable angina (38.8%), or STEMI (22.4%) [89]. Relatedly, faster activation of the cath lab was associated with improved reperfusion times (98 versus 135 min) for primary PCI versus delayed activation and lower in-hospital mortality (2.8% vs. 3.4%; *p* = 0.01) versus no activation [90,91]. For patients with STEMI, expedited cath lab preactivation is critical in reducing PCI-related delays.

PCI is among the most performed medical procedures [80], particularly in complex cases [92], and is a target for bundled payment initiatives. However, readmissions after PCI are common and costly. Data from the U.S. Nationwide Readmission Database revealed that ~25% of patients who undergo PCI had unplanned readmissions within six months; the time at which patients are at greatest risk is seven days post-discharge [93]. Readmission payments were also the primary driver of variation in 90-day bundled episode payments after PCI in 33 Michigan hospitals (46.2%), followed by post-acute care (22.6%) [94]. Similarly, 74 to 93% of the variation in post-acute Medicare spending in the U.S. for acute myocardial infarction was primarily driven by readmissions to skilled nursing facilities [95]. Thus, minimizing the likelihood of PCI readmissions holds substantial promise for improving value in cardiology care and the success of bundled payments [96].

An SLR evaluating causes for 30-day readmissions after PCI demonstrated that reinfarction/stent thrombosis (2.5–9.5%), heart failure (5.9–12%), chest pain (6.7–38.1%), and bleeding (0.7–7.5%) are key reasons [97]. These unplanned readmissions are associated with high costs compared to not readmitted patients, with the index admission cost being USD 18,631 (readmission between 0 and 7 days) to USD 23,797 (readmission between 91 and 180 days) [93]. Further, costs for patients experiencing chest pain, angina, or ACS were 1.8 times greater (USD 32,437 vs. USD 17,913, *p* < 0.001) than for those who did not experience these symptoms at one year in a U.S. multipayer administrative claims database of patients with incident inpatient PCI admissions [98]. For chronic CAD patients, a U.S. review found that 12.4% of patients at a 2-year follow-up experienced MACE, resulting in USD 48,457 higher multivariate-adjusted healthcare costs than for patients who did not experience MACE [99]. However, early discharge (3 days) after PCI is associated with decreased 30-day readmission rates and costs in the U.S. [100], while same-day discharge leads to cost savings without compromising procedural outcomes [101,102] in patients presenting with chronic coronary syndrome.

Relatedly, approximately 15–30% of patients presenting with their first myocardial infarction will be rehospitalized for recurrent cardiac events [103,104,105,106]. Patients hospitalized for recurrent ACS typically have a higher prevalence of diabetes, hyperlipidemia, hypertension, and vascular disease compared to patients experiencing only an incident event [107]. They also more often suffer from suboptimal treatment before index hospital admission [106]. Data from the French MONICA population-based registries showed that mortality rates at 28 days (9% vs. 5%, respectively) and one year were higher among recurrent versus incident cases (14% vs. 8%, respectively), independent of age and sex. The higher one-year mortality rate observed among recurrent cases was explained by older age, comorbidities, and worse cardiac function, emphasizing the need to reinforce secondary prevention after an ACS and thus optimize persistent risk factors [106] and patient compliance. In a report from the TRACE-CORE study, persons with recurrent ACS were more likely to report anxiety, depression, stress, and lower mental and physical quality of life. These individuals were also more prone to cognitive impairment than individuals with ACS for the first time [108]. 

Additional burdens associated with CAD treatment using PCI are incomplete revascularization (revascularization restricted to the culprit artery) and in-stent restenosis, a renarrowing of the blocked section of the coronary artery after stent deployment, which can negatively impact patient survival [109]. Incomplete revascularization leads to worse clinical outcomes for patients and was more frequently observed in complex patients, likely based on patient clinical characteristics (e.g., advanced age, comorbidities), lesion characteristics (e.g., chronic total occlusions, CTO), and failed primary PCI [110]. Operator choice may also be a factor [110], as high-volume operators who have been in practice longer have lower rates of incomplete revascularization in patients undergoing PCI [111]. The evidence shows that acute and chronic CAD patients who underwent PCI and experienced incomplete revascularization had significantly higher MACE rates, odds of death, and repeat revascularization than patients with complete revascularization [110,112]. Longer-term follow-up studies revealed that for PCI, the degree of incompleteness was associated with greater 10-year mortality than those undergoing PCI with complete revascularization (50.1% vs. 22.2%; adjusted hazard ratio, 3.40 [95% CI, 2.13–5.43]) [113]. For in-stent restenosis, retrospective data from the CathPCI registry between 2009 and 2017 demonstrated that this burden accounts for ~10% of all PCI procedures, with ~25% of patients presenting with myocardial infarction [114]. Drug-eluting stents have received a Class IA AHA recommendation to be used in preference to bare-metal stents to prevent restenosis, myocardial infarction, or acute stent thrombosis in patients undergoing PCI [83]. 

In stable CAD, recommendations for PCI are limited [83], yet it is often used in this patient population. In a U.S. registry of patients undergoing nonacute PCI, 3.3% of PCIs were classified as rarely appropriate when using the current appropriate use criteria (AUC) for PCI, with rates increasing to 22.3% when incorporating data from the COURAGE and ISCHEMIA trials [115]. Of note, approximately one in six patients was asymptomatic at the time of PCI [115], indicating that efforts to improve patient selection are needed. A U.S. study found that a higher percentage of inappropriate PCI procedures were often performed in higher volume hospitals (>400) and by higher volume operators (>200) than their lower volume counterparts [116]. These findings may be partly attributed to physician reassurance, patient factors (e.g., fear of another cardiac event, unquestioned acceptance of prescheduled procedures), and financial drivers in a fee-for-service healthcare system [117,118]. Notably, the New York State Department of Health announced the intention of withholding reimbursement for Medicaid patients with inappropriate PCI [119]. As such, all inappropriate PCIs decreased from 18.2 (2010) to 10.6% (2014) (from 15.3 to 6.8% for Medicaid patients) [119]. 

### 3.4. Post-Acute Care—Challenges with Infrastructure, Access, and Adherence

Patients with acute CAD continue to be at risk for future ischemic events following revascularization (e.g., with PCI); therefore, secondary prevention is essential for improving patient outcomes and preventing further damage and disease progression. Cardiac rehabilitation is a critical component of secondary prevention and includes exercise training, psychosocial and weight management, and tobacco cessation to reduce repeat hospitalizations and improve cardiovascular mortality and quality of life [120,121,122]. A complete course of cardiac rehabilitation in the U.S. is typically ≥36 supervised sessions over 12 weeks, although this can vary [123]. However, cardiac rehabilitation remains underutilized, with low participation and adherence rates. A meta-analysis showed participation rates of <50% in most countries, and documented dropout rates are up to 56 and 82% in high- and middle-income countries, respectively [124]. Challenges include suboptimal referral rates, limited access, language barriers, low motivation/low self-efficacy, and challenges in the patient–provider relationship, amongst others [125]. To ensure optimal utilization of cardiac rehabilitation, information (i.e., patient history and clinical documentation), management (i.e., ease of referral process), and relational continuity (i.e., consistent staffing) are recommended [28]. 

A 2019 study revealed cardiac rehabilitation is available in only half of the nations around the world, and the geographical distribution of these programs is negatively correlated with the incidence of CAD [124,126,127]. Furthermore, a minority of eligible patients have participated in cardiac rehabilitation over the past decade, suggesting referral rates should be improved [127]. Strategies include automatic referrals at discharge, strong coordination among inpatient, home health, and outpatient cardiac rehabilitation programs, and patients’ medical teams and families supporting and encouraging participation [127,128]. Maintaining high standards of post-acute care for treated patients can also be challenging, especially with the lack of follow-up by healthcare professionals. 

Proper continuity of care from hospital to home is essential as patients require lifestyle adjustments, including incorporating new medications and acquiring support [129]. A Dutch study found that participation in a multidisciplinary outpatient cardiac rehabilitation program for CAD was associated with a 32% lower mortality risk [130]. Additionally, hospital readmissions may be prevented through clinical interventions that target post-procedure vulnerabilities. A U.S. study used a validated questionnaire to identify patients at high risk for readmission after PCI and subsequent targeted interventions, which resulted in an absolute decline in readmissions from 9.6 to 5.3% over four years at a large tertiary care facility [131]. This approach also helped educate patients about chest discomfort through videos and implemented a risk stratification algorithm to triage patients presenting with chest pain after PCI [131]. Furthermore, a qualitative study found that information related to self-management was scarce when CAD patients (including those who underwent PCI) communicated with their physicians during visits, suggesting that increased patient–provider communication may improve post-acute care [132]. A study found that cardiovascular rehabilitation referral was related to greater patient–provider interactions (OR = 2.82, 95% CI = 1.01–7.86) and less patient concern and worry (OR = 0.64, 95% CI = 0.45–0.89) [133]. For patients post-myocardial infarction undergoing cardiac catheterization, a hybrid program using a family-centered model empowers the patient/family unit to promote health quality and improved quality of life over time versus traditional home cardiac rehabilitation, suggesting that this approach is effective for increasing uptake [134]. 

### 3.5. Additional Challenges—Clinician Burnout 

PCI is performed in the cath lab, which is prone to operational inefficiencies [13,14], distractions [15], and infrequent use of closed-loop communication [16]. Operational inefficiencies may include a lack of an electronic scheduling system, decreased utilization of patient preparation and recovery areas, and communication about room and patient availability [13]. In turn, late arrival times, delayed patient preparation, physician readiness, delays in procedure or equipment set-up, and inadequate staffing contribute to suboptimal patient flow [17]. Data from a prospective observational study indicate that procedural distractions (e.g., interruptions) occurred in 55% of cases (PCI, standby coronary angiography, CTOs) in the cath lab [15]. Operators reported higher cognitive and physical workload and effort levels during cath lab cases in which distractions occurred [15]. These factors impact quality improvement in the cath lab, clinician workload, patient flow, and safety. 

Professional burnout in the field of cardiology is prevalent, with substantial sustaining impacts on patients and the healthcare system. More than a quarter of U.S. cardiologists and fellows-in-training reported feeling burnt out, and almost 50% of the remainder stated that they were stressed [26]. These rates have only increased from prepandemic levels [135]. A population-based survey found that U.S. physicians experiencing burnout had more than twice the odds of self-reported medical errors [136,137], which are associated with physical and emotional impacts, loss of trust, and avoidance of healthcare [138]. Clinician burnout can also lead to decreased patient satisfaction, increased disruptive behavior, a loss of professionalism, and a decreased level of empathy [139,140]. Drivers associated with burnout include a lack of control over workload, a hectic work environment, a nonpredictable schedule, and irregular and long working hours [139,140]. These ramifications may result in loss of staff, as a U.S. study reported turnover for critical care registered nurses was greater than the national average at 27.5% in 2021 versus 18.7% in 2020, partly due to workload, working conditions, and scheduling [141]. Furthermore, interventional cardiologists are prone to suffer from acute and/or chronic sleep deprivation, which has been associated with impaired performance, cognitive deficits, workplace errors, and injuries [142]. A survey of 6683 cardiovascular workers revealed that 25% of respondents had a sleep disorder, with the leading cause being work (66%) [142]. If left unaddressed, sleep deprivation may increase the risk of adverse health outcomes for clinicians and their patients. Furthermore, inefficient use of health information technology, such as electronic health records (EHRs), creates documentation burdens contributing to burnout [143]. Of note, cardiologists maintain irregular and hectic work schedules, which are exacerbated by documentation requirements [27]. In fact, many U.S. physicians, including cardiologists, have reported increased time dealing with documentation and less time interacting with their patients. As such, improvements in documentation and exploring practice redesign may help alleviate stress and address clinician burnout to ensure high-quality patient care [27].

### 3.6. Additional Challenges—Technology Risks

Patients are also impacted by the challenges and inefficiencies occurring within CAD emergent, diagnostic, and interventional care, as some technologies can contribute to increased patient risk. Accurate imaging of coronary arteries during cardiac catheterization depends on the intravascular injection of iodinated contrast media and fluoroscopic imaging. As such, patients may be exposed to a significant amount of contrast media and both patients and clinicians to ionizing radiation [144,145]. Radiation exposure from fluoroscopic imaging is associated with acute and chronic tissue injuries, as well as long-term cancer risk [144,145,146]. Furthermore, contrast media administration during angiography and PCI can increase the risk of contrast-induced acute kidney injury, an impairment of renal function reported in up to 10% of cardiac catheterization and PCI patients [145,147]. Patients with ACS have about a three-fold risk of developing contrast-induced acute kidney injury versus those without ACS [148]. This impairment is associated with short- and long-term mortality and MACE [149,150]. As aging CAD patients are driving an increase in complex PCI, there is a need to implement proper protective and preventative measures for patients and clinicians. 

Advancements in technologies to improve detection, diagnosis, and treatment are considerably expanding; however, for clinicians, complex technologies require a mastery of set-up, operation, and troubleshooting, introducing risk and resulting in a high training burden [151]. While modern CTO PCI techniques improve patient symptoms [152,153], they are technically demanding and require operator experience and training to ensure procedural success [154]. A report from the U.S. National Cardiovascular Data Registry revealed that less operator experience was associated with a lower likelihood of procedural success (59% vs. 96%, *p* < 0.001) and higher MACE (1.6% vs. 0.8%, *p* < 0.001) in patients undergoing CTO PCI versus non-CTO PCI patients [155]. Although highly adopted in Europe, the transradial PCI technique has lagged in the U.S. due to a later generation of clinical data and the learning curve. Similarly, IVUS and optical coherence tomography (OCT) for intravascular imaging PCI have also not been consistently adopted, partly due to a lack of training, the associated learning curve [156], costs, and a lack of adequate reimbursement. Expertise is required to guide PCI using IVUS, a process prone to bias and human error [157], while image acquisition using OCT can be suboptimal without proper education and training [158]. Implementing a standardized and streamlined image acquisition protocol and efficient training with complex technologies would help minimize errors in image interpretation, optimize procedural results, and improve patient outcomes. However, time constraints can preclude addressing risks, learning objectives, and learning curves, creating a poor environment for product use. As a result, some patients who could benefit from the technology do not receive it or do not benefit fully [159]. 

## 4. Evolving Solutions

Across the CAD pathway, the burdens associated with access, testing accuracy, appropriate use, patient complexity, clinician burnout, and technology use can negatively impact workflow, decision-making, and patient care. The COVID-19 pandemic has exacerbated the inadequacies in access and highlighted the need for intensified triage and virtual care to facilitate the timely evaluation of CAD patients without unnecessary travel to hospitals or outpatient clinics [160]. In turn, staff can disseminate, educate, and improve CAD diagnosis using remote proctoring while conserving limited resources [161]. Improvements to the CAD pathway can be implemented beyond the COVID-19 pandemic to minimize the disruptions caused by reduced access to diagnostic testing and procedures [23]. 

**Figure 1:**
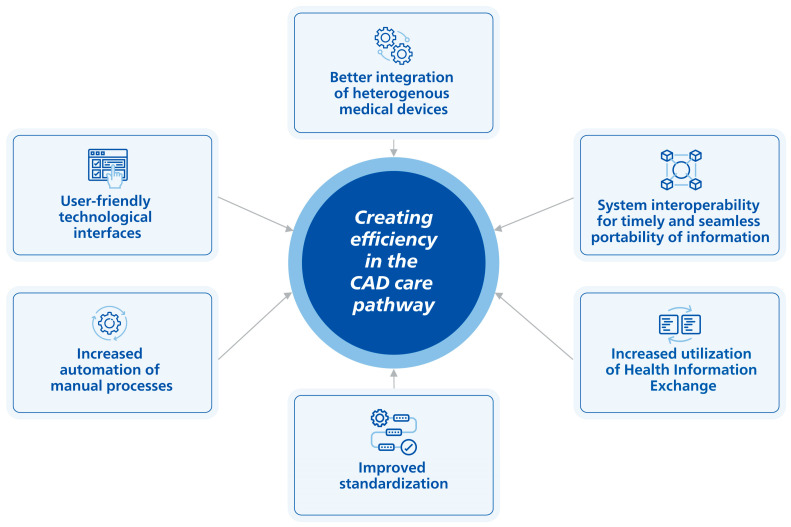
Critical success factors to address inefficiencies in the CAD care pathway: (1) better integration of heterogenous medical devices (i.e., integrated systems with digital controls to dynamically manage multiple devices and improve workflow); (2) enhanced system interoperability (i.e., interoperable medical devices that effortlessly communicate with other devices through a common language to reduce errors); (3) increased utilization of health information exchange (i.e., quick and efficient transfer of critical medical information/tests); (4) improved standardization (i.e., standardized documentation templates for structured reporting to decrease workload); (5) increased automation of manual processes (i.e., automated processes can facilitate image interpretations to improve confidence and accuracy); (6) user-friendly technological interfaces (i.e., easily accessible web-based interfaces in the catheterization lab that enhance the user experience). CAD: coronary artery disease.

In addition to virtual care, advances in technology for diagnosis and treatment, the automation of processes, and integrated care are needed to alleviate the challenges and burdens identified within the CAD care pathway. This is critical as technologies, including medical devices, are typically heterogeneous and were not constructed to automate clinical workflows, streamline communication, and improve efficiencies [162,163]. Table 4 presents an overview of the burdens and inefficiencies in the CAD care pathway and the impacts on patients, providers, and the healthcare system. The complexity of CAD management requires more efficient and high-quality clinical solutions that evolve to improve the user experience and help address the burden of disease for patients and providers over time (Figure 1). A significant part of helping improve patient outcomes relies on integrated diagnostic and treatment solutions that harness the use of information technology to seamlessly connect, provide automated assistance, offer standardization, deliver real-time data availability, and offer a user-friendly interface to improve workflow and ensure exceptional care [164]. However, implementing medical technologies within healthcare is a complex and arduous process [165]. Considerable effort is needed for hospitals to adopt new technological solutions, as the diffusion and implementation of technology are highly influenced by multiple factors, including technology-specific challenges, the performance and efficacy of the device, reimbursement, and characteristics of the adopters, amongst others [165]. At the same time, hospitals and health systems have continually faced a range of financial and operational challenges (e.g., staff shortages, increasing costs, medical supply issues, etc.) that may also impact the uptake of these solutions [166]. Although technology can utilize the six critical success factors discussed below, there remain continuous advancements to optimize these technologies to meet the changing needs of healthcare. Furthermore, the life expectancy of imaging equipment is based on utilization but is reported to be between 8–12 years for CT, MRI, SPECT, and interventional angiography, and 7–9 years for ultrasound, according to the Canadian Association of Radiologists [167]. Older equipment may lead to crucial delays in the diagnosis and treatment of the patient. Additionally, older equipment is limited by its inability to communicate in an up-to-date environment which requires a performing electronic infrastructure, such as teleradiology, and connection with the electronic patient record [167]. The European Society of Radiology (ESR) states that imaging equipment that is up to 5 years old has state-of-the-art technology [167]. The ESR is promoting the use of up-to-date equipment and recommends that at least 60% of the installed equipment in radiology departments be less than 5 years old, up to 30% should be 6–10 years old, and not more than 10% should be older than 10 years [168]. However, this recommendation is not met in most European countries for cardiovascular imaging [168]. Due to the prolonged use of equipment, it may take time before new technology to improve efficiency is fully adopted in hospitals.

Inefficient communication is a prevalent organizational challenge in healthcare and may have several potentially negative consequences for both patients and clinicians. Inadequate communication has been shown to compromise the continuity of care and patient safety, as well as increase worker burden and treatment delays [169,170]. Furthermore, communication inefficiencies in hospitals generate an estimated excess of USD 800 million per year [169,170].

Integrated systems with digital controls to manage multiple devices or systems dynamically have been developed to improve communication, workflow, and efficiency, and clinicians appreciate those that are easy to learn, use, and troubleshoot [151,163]. Relatedly, interoperability allows different information systems and devices to access, exchange, integrate, and cooperatively use data to provide timely and seamless portability of information [171]. This is critical for reducing medical errors by making the report readings available in real time and directing test results to clinicians in a timely and precise manner [172]. The evidence suggests that several of the most common causes of medical errors, including drug and diagnostic errors and failure to prevent injury, could be mitigated by improved medical device interoperability [173,174].

The integration of EHRs and electronic medical records (EMRs) enhances communication capacity and information flow across the continuum of care [175] and provides relevant information in real time [176]. Accessing information remotely can help to facilitate seamless communication between care providers and patients [175]. Health information exchange improves the quality and efficiency of patient care by increasing provider access to patients’ medical histories [177]. In a longitudinal cohort study of patients in New York State who underwent imaging procedures, health information exchange use resulted in an overall estimated annual saving of USD 32,460 in avoided repeat imaging [178], and it can also be used to hasten the transfer of urgent medical information. For instance, an automated, interoperable 12-lead ECG mobile alert system successfully transmitted alerts (94% of cases) to physicians within five minutes via a mobile application and patient EMRs, resulting in higher rates of admission and cardiac-related diagnosis in the “alert” versus “non-alert” groups [179]. Similarly, real-time analysis and classification of ECG signals can aid in CAD diagnosis, especially in intensive care units where the real-time monitoring of patients is critical [180]. Regarding patient outcomes, Florida hospitals participating in HIE versus those not participating were associated with a decrease in the probability of unplanned 30-day readmissions for acute myocardial infarction, with the sharing of radiology reports having the greatest effect on reducing readmissions [181]. Since reducing hospital readmission is a priority in CAD, implementing interoperable health information technologies is necessary to improve patient care [181]. 

A variety of evidence identifies the value of device integration and intuitive interfaces. To address workflow inefficiencies in the cath lab [13,14], an integrated interventional suite means saving time and personnel because more procedures can be completed in the same room by the existing staff and without relocating equipment or personnel from another area [182]. In addition, an integrated suite helps manage complex patients by simplifying certain procedures and improving technological capabilities, including higher-quality images and fast graphic abilities [182]. User-friendly and intuitive interfaces also improve procedural efficiencies. By implementing a web-based interface in the cath lab as part of a quality improvement initiative, procedures started an average of 17 min earlier, and turnaround times were reduced from 20.5 to 16.4 min compared to before the program could be documented [13]. 

Furthermore, the evidence base for advancements in technology is growing exponentially with the development of solutions that enhance precision in the detection and risk stratification of CAD, as well as improved treatment success. Advances in image processing and computer hardware and software have facilitated the development of automated visualization methods that use less contrast to guide PCI, thereby improving procedural confidence and risk [183,184,185]. Advanced and innovative technologies, including IVUS, iFR, and FFR that have recently received Class IA AHA recommendations, can help improve the limitations associated with ICA and patient outcomes and improve procedure efficiency [20,83,186]. Methodologies have been developed that operate with an accessible interface and can process intravascular imaging data, reliably quantify lesion severity, and coregister intravascular and X-ray angiographic data to comprehensively assess plaque distribution and guide PCI [157]. The localization of IVUS images with automated angiography coregistration can also help to reduce the risk of geographic mismatch (residual disease at the stent edge), which is associated with an increased risk of adverse events after PCI [187,188], and help address operator learning curves [184]. Other approaches using near real-time, fully automated technologies for accurately identifying, detecting, and quantifying luminal borders in intravascular images using IVUS show feasibility for clinical use versus manual analysis [189]. 

The evidence also supports automation and standardization for improving clinician reporting and image interpretation. For instance, natural language processing methods using machine learning transform clinical text into structured data [190]. Structured reporting integrates workflow and documentation processes and achieves data interoperability among information technology systems to maximize accuracy, completeness, and efficiency [191]. Well-designed checklists help with standardization and reduce errors in routine care and emergencies [192,193,194,195]. For cardiac imaging, structured reporting can decrease errors and standardize how findings are communicated [196,197]. For instance, the CCTA workflow is laborious, with an average post-processing and reporting time of ≥30 min [198]. A prospective study of patients with chest pain undergoing CCTA found that structured reporting platforms with the automated calculation of the Coronary Artery Disease Reporting and Data System (CAD-RADS) scores outperform manual classification by preventing human errors, improving data quality, and supporting the standardization of clinical decision-making [199]. For CCTA, a retrospective study found that a deep learning-based algorithm helped streamline CCTA reconstruction and interpretation workflows for CAD patients, significantly improving time efficiency and diagnostic consistency [200]. Deep learning algorithms for the automated interpretation of echocardiographic images offer the opportunity to remove the burden for highly trained individuals to conduct manual image analysis [201] and may eliminate some of the intensive training and skill maintenance required of operators [202] and reduce human error [203].

## 5. Conclusions and Future Directions

Although advances in diagnosis and treatment have improved patient outcomes, CAD remains the leading cause of mortality worldwide and is expected to increase in the future. PCI cases continue to grow in complexity due to the growing prevalence of comorbidities, including obesity, type two diabetes, and increasing age. The rapid evolution of advanced technologies in recent years has helped to improve patient care, boost workflow efficiencies, and enhance clinician confidence. However, significant inefficiencies related to diagnosis, treatment, and patient flow across the continuum of care are associated with financial and clinical burdens. Innovative, integrated diagnostic and treatment solutions that are interoperable and provide automated assistance are fundamental to improving clinical efficiency and ensuring exceptional in-person and remote patient care. This narrative review highlights the main areas that require further research to address the challenges and burdens faced by CAD patients and their caregivers from varying geographic regions and healthcare systems, as well as an opportunity to discuss efficient and cost-effective solutions for disease management.

## Figures and Tables

**Table 1:** Key components of the CAD care pathway.

Primary Prevention	Emergency Care	Diagnosis	Treatment	Secondary Prevention	Follow-Up
Risk assessment	ECG	Anatomical testing (e.g., CCTA, ICA)	PCI	Cardiac rehabilitation	Incorporating new medications
Medical history	Risk stratification	Functional testing (e.g., stress ECG, SPECT)	CABG	Repeat revascularization or surgery (if required)	Lifestyle support
Physical examination	Medical therapy	Biomarker testing (e.g., cardiac troponin)	Guideline-directed medical therapy and risk factor control		Risk factor education
Laboratory blood tests					
Medical therapy					

CABG: coronary artery bypass graft surgery; CAD: coronary artery disease; CCTA: coronary computed tomography angiography; ECG: electrocardiogram; ICA: invasive coronary angiography; PCI: percutaneous coronary intervention; SPECT: single photon emission computed tomography.

**Table 2:** Search terms/overview of search strategy.

Research Theme	Key Search Terms Used
General	(coronary artery disease [tw] **or** catheterization lab [tw] **or** cath lab [tw] (cardiologist [tw] **or** cardiologists [MeSH])) **AND**
Overview of coronary artery disease pathway	(review [tw] **or** systematic review [tw]) **AND**
Diagnostic burdens	(diagnostic error [tw] **or** delay [tw] **or** wait times [tw] **or** (suboptimal diagnosis [tw] **or** diagnostic challenges [MeSH]) **or** accuracy [tw])) **OR**
Treatment burdens	(undertreatment [tw] **or** (incomplete [tw] **or** treatment gap [MeSH]) **or** (delay [tw] **or** wait time [MeSH]) **or** overuse [tw] **or** underuse [tw]) **OR**
Workflow efficiencies	(workflow [tw] **or** ((in)efficiency [tw] **or** productivity [MeSH]) **or** turnover time [tw] **or** procedure time [tw] **or** electronic medical record [tw] **or** integration [tw] **or** standard * [tw] **or** streamline [tw] **or** error [tw] **or** administrative [tw] **or** (user-friendly [tw] **or** ease of use [MeSH]) **or** automat * [tw] **or** data integration [MeSH] **or** artificial intelligence [tw] **or** data access [tw] **or** real-time data [tw] **and** (interoperability [tw] **or** (integration [tw] **or** integrated [MeSH]))) **OR**
Post-acute care	(rehabilitation [tw] **or** follow-up [tw]) **OR**
Healthcare worker burdens	((healthcare worker [MeSH] **or** healthcare provider [MeSH] **or** technologist [tw] **or** clinician [tw] **or** nurse [tw] **or** cardiologist [tw]) **and** (stress [tw] **or** burnout [tw] **or** satisfaction [tw] **or** workload [tw] **or** radiation [tw] **or** learning curve [tw]) **OR**
Economic burdens	(cost [tw] **or** economics [tw] **or** cost-effectiveness [tw] **or** economic burden [tw]) **OR**
Patient burdens	(contrast [tw] **or** radiation [tw] **or** (acute kidney injury [tw] **or** complication [tw] **or** readmission [tw]))

Note: all searches were targeted in combination with the general search (i.e., coronary artery disease); the asterisk (*) was added to the root of the word to instruct the database to search for all forms of the word; supplemental search terms: interoperability, integrated solutions/device, DICOM, connectivity, digitization, DICOM: digital imaging and communications in medicine, MeSH: medical subject headings, tw: text word.

**Table 3:** Strengths and limitations of selected diagnostic methods for patients with suspected CAD.

Modality	Strengths	Limitations
***Functional testing***		
Stress ECG	Noninvasive Low cost, efficient to perform Functional capacity can be estimated Widely available (e.g., point of care)	Limited accuracy Limited ability to localize ischemia Minimal detection of single-vessel disease Operator and patient dependence Limited by patient factors
Stress Echo	Noninvasive No radiation Can identify structural information and localize ischemia Real-time imaging of cardiac function	Suboptimal performance in certain patients (e.g., obesity, pulmonary disease) Required operator skill Mostly qualitative analysis Suboptimal sensitivity for single-vessel disease Limited by patient factors
SPECT	Relative perfusion evaluation (relative) Quantitative analysis possible Comparable performance for exercise and pharmacological stimuli	Radiation exposure (12–37 mSv) False negatives resulting from “balanced ischemia” Limited by patient factors
Stress PET	Absolute quantitation of perfusion defect Greater image quality versus SPECT	Less available Requires pharmacological stimuli Costly Radiation exposure (10–14 mSv) Limited by patient factors
Stress CMR	High resolution Subendocardial perfusion No radiation Can identify structural information Qualitative and semi quantitative analyses possible	Less available Costly Claustrophobia, arrhythmias, and adiposities can be limiting
***Anatomical testing***		
CCTA	Noninvasive and widely available Identifies obstructive CAD Rapid structural assessment Can evaluate CT-FFR	Unable to confirm ischemia Susceptible to motion artefacts Calcification can restrict lumen assessment Laborious interpretation and image construction Radiation exposure (1–5 mSv) Contrast medium can be limiting in patients who are unable to tolerate it
Spectral detector CT	Conventional and spectral data simultaneously Virtual calcium score calculation Used for reduction in calcium blooming and improve lumen definition Reduced radiation dose and lower contrast medium use than CCTA ^2^	Requires a tube potential of at least 120 kVp Contrast is lowered in conventional images ^3^ due to higher kVp ^3^ Limited by patient factors
ICA	Considered the gold standard Revascularization can occur at the same sitting Can be combined with invasive functional assessment (FFR, iFR)	Unable to independently confirm ischemia or amount of luminal narrowing Radiation exposure (7.60–17.8 mSv) ^1^ Invasive with risk of complications (1 in 1000 of MI, stroke, or death) Specialized equipment and trained staff Limited by patient factors

CAD: coronary artery disease; CCTA: coronary computed tomography angiography; CMR: cardiac magnetic resonance; CT-FFR: computed tomography fractional flow reserve; ECG: electrocardiogram; FFR: fractional flow reserve; iFR: instantaneous flow reserve; ICA: invasive coronary angiography; PET: positron emission tomography; SPECT: single photon emission computed tomography. Source: modified from [6]; ^1^ interquartile range from [54]; ^2^ [55]; ^3^ [56].

**Table 4:** Overview of burdens and inefficiencies in the CAD care pathway and key impacts.

*Burden*	*Key Impacts on the Healthcare System, Providers, and Patients*
**Suboptimal diagnosis (e.g., false negatives)**	Higher rates of misdiagnosis among women Fatal acute MIs Higher rates of recurrent ischemia Higher incidence of MACE Costs (testing costs, hospital costs, productivity loss)
**Delays in accessing emergent diagnostic care**	
**Increasing complexity in clinician decision-making with expansion of test strategies and options**	Significant uncertainty on how to approach a diagnosis Ineffective functional testing (e.g., low CAD prevalence setting) Wastage of resources with inappropriate tests Potential increased risk of MACE
**Overuse of invasive technologies (e.g., inaccuracies, false positives)**	Unnecessary radiation exposure Nonobstructive and asymptomatic patients crowding procedure rooms Patient discomfort and procedure-related risks
**Underuse of emergent PCI**	Longer time to treatment Potential increased risk of mortality and MACE
**Increasing use of PCI in complex cases and associated burdens**	Increased costs due to hospital readmission Rising costs due to higher prevalence of PCI complications Recurrent cardiac events Incomplete revascularization and in-stent restenosis Lower patient quality of life Higher patient psychosocial burden
**Overuse of PCI**	Increase in costs Increased patient risks
**Underutilization of cardiac rehabilitation**	Repeat hospital readmissions Lower patient quality of life Higher mortality risk
**Inefficiencies in the catheterization lab**	Increased physical and mental burden on clinicians Compromised patient safety Disruptions in communication pathways Coordination issues in hospital operations Inefficient workflow
**Clinician burnout and sleep deprivation**	Increased chance of medical errors and injuries Decreased clinician professionalism
**Manual processes**	Increased documentation requirements Less provider–patient interaction
**Technology risks**	Radiation exposure and associated risks Contrast-induced acute kidney injury in patients High training burden and learning curves with complex technology Decreased procedural success and potential risk for MACE Increased costs and medical errors among improperly trained clinicians
**COVID-19**	Reduced or delayed admissions of cardiac patients Deferred cardiac procedures Pandemic-related clinician burnout Inequitable access to treatment among patients

CAD: coronary artery disease; MACE: major adverse cardiac event; MI: myocardial infarction; PCI: percutaneous coronary intervention.

## Data Availability

The data underlying this article are sourced from the public domain and are available in the articles cited throughout.
